# A muscle exercise research revolution powered by -omics at single cell and nucleus resolution

**DOI:** 10.1186/s12915-023-01781-1

**Published:** 2023-12-29

**Authors:** Kevin A. Murach, Charlotte A. Peterson

**Affiliations:** 1 Exercise Science Research Center, Molecular Muscle Mass Regulation Laboratory, Department of Health, Human Performance, and Recreation, University of Arkansas, Fayetteville, AR USA; 2 Center for Muscle Biology, College of Health Sciences, University of Kentucky, Lexington, KY USA

## Abstract

**In the last few years, single cell and single nucleus RNA-sequencing technologies have been leveraged in exercised adult skeletal muscle to reveal complex cell type-specific regulation of muscle plasticity. Expanding -omics technology at single cell resolution, complimented by advanced computational approaches, promise to rapidly accelerate our understanding of how exercise confers its numerous beneficial effects, paving the way for cell type-specific and targeted therapeutics to increase exercise responses during aging and in the face of chronic disease.**

## Molecular underpinnings of muscle adaptation

Molecular exercise research leapt forward when transcriptome profiling technology was applied to skeletal muscle samples. In 2000, Jozsi et al. utilized “low-density” microarrays (~ 500 genes) to provide the first broad transcriptomic characterization of the acute response to a bout of resistance exercise in human skeletal muscle [1]. A few salient observations emerged from this investigation: (1) the gene expression response to exercise is attenuated in older versus younger men consistent with lower adaptive potential when aged, (2) an inflammatory transcriptional signature characterizes unaccustomed exercise, and (3) genes that may be enriched in non-muscle mononuclear cells (e.g., endothelial cells and macrophages) are altered by a bout of resistance exercise [1]. The gene expression changes that occur following a bout of exercise in muscle provide a lens into eventual long-term adaptive remodeling, as mRNA can be translated into protein that ultimately dictates cellular phenotype and function. Studies such as Joszi et al.’s laid the foundation for understanding the molecular underpinnings of the muscle adaptive process throughout the lifespan.

## Whole muscle tissue transcriptomics with exercise

Numerous muscle research advancements have emerged in the past 20 + years, including the application of RNA-sequencing (RNA-seq) to exercised muscle samples. RNA-seq provides broad-spectrum coverage of the protein-coding transcriptome, as well as splicing variants and non-coding RNA species. Developments in high-density microarray technology (> 20,000 genes) have also permitted wide-ranging coverage of the transcriptome. Collectively, both can provide a fairly comprehensive picture of transcriptome changes in muscle. A large number of published human skeletal muscle RNA-sequencing and array exercise transcriptome data were recently compiled into a meta-analysis database called MetaMex that is freely available for online interrogation [2]. This tool can be leveraged to understand transcriptome responses to acute and chronic endurance, resistance, and combined (concurrent) exercise across different ages, sexes, and post-exercise recovery time points. With this tool, changes to the expression of numerous genes across exercise modes were found to contribute to exercise adaptation. One such highly responsive gene, nuclear receptor subfamily 4 group A member 3—*NR4A3*—is induced by any form of exercise and regulates muscle metabolism [2]. As the largest cells by volume in muscle, multinuclear myofibers understandably contribute significantly to the whole muscle tissue transcriptome in response to exercise. Until recently, though, the extent to which mononuclear cell types in muscle such as muscle stem cells (satellite cells), fibro-adipogenic progenitors (FAPs), endothelial cells, immune cells (e.g., macrophages), and minority cell populations interact with and contribute to human exercise responses, as well as adaptive heterogeneity, has not been possible. The emergence of single cell RNA-sequencing (scRNA-seq) and single nucleus RNA-sequencing (snRNA-seq) are the catalysts for defining the breadth and complexity of cellular choreography in muscle during exercise adaptation.

## The age of single cell and nucleus RNA-sequencing with exercise in skeletal muscle

In 2018, a *Tabula Muris* of 20 mouse organs using scRNA-seq defined the first cellular atlas of skeletal muscle mononuclear cells [3]. This information is foundational because it (1) reinforced the extent of cellular diversity in skeletal muscle outside of the myofiber and (2) charted a path forward for using emerging single cell technology in skeletal muscle. In 2021, the first study leveraging scRNA-seq in the context of adult skeletal muscle adaptation to hypertrophic mechanical loading (i.e., resistance exercise mimetic) in mouse was published [4]. Satellite cells are the bona fide muscle stem cell population in skeletal muscle. This study provided initial evidence that satellite cell-derived myonuclei that fuse into myofibers during hypertrophy may differ from resident myonuclei. This work also expanded on how satellite cells communicate widely to other muscle-resident cells, such as FAPs, to control extracellular matrix (ECM) remodeling, inflammatory-related gene expression, and potential cell fate. The evidence suggests that intercellular communication from satellite cells can be mediated by extracellular vesicles (EVs) that deliver miRNAs such as miR-206 to target recipient cell mRNAs and affect cellular function [4]. Intercellular communication by satellite cells may help create a permissive environment for sustained myofiber growth during loading [4]. Using snRNA-seq (which contains myonuclei as well as nuclei from mononuclear cells in muscle) combined with progressive weighted wheel running (PoWeR) in mice, we further defined the role of satellite cells in the exercise training response [5]. Satellite cells positively influenced oxidative metabolism, ribosome biogenesis, and myofibril assembly related gene expression in myonuclei after 4 weeks of high-volume PoWeR, which was characterized by oxidative fiber type transitioning and hypertrophy [5]. Additional analysis suggested that (1) satellite cells activate but do not necessarily fuse during adult muscle adaptation to exercise, and (2) satellite cell depletion dysregulates myonuclear gene expression and causes “cryptic,” or transcriptionally ambiguous, myonuclei to emerge [6].

It is intuitive that the first studies using sc- and snRNA-seq focused on the influence of satellite cells on hypertrophic muscle adaptation given their myogenic potential. Additional scRNA-seq studies have since provided more holistic information on the function of various muscle mononuclear cells, as well as myofibers, during adaptation to exercise. Expanding on the findings of Joszi et al. > 20 years prior, work in 2021 using bulk RNA-seq in human muscle following resistance training and scRNA-seq in mouse in response to mechanical overload highlighted the role of macrophages in ECM remodeling [7]. This study collectively revealed that myofiber-derived leukemia inhibitor factor (*LIF*) specifically altered macrophage gene expression of the ECM remodeling gene matrix metalloproteinase 14 (*MMP14*) during hypertrophy to influence collagen turnover. Physical activity in mice has also been demonstrated to specifically target macrophages in muscle. scRNA-seq showed that chronic unweighted wheel running normalized dysregulated inflammatory gene expression in macrophages of aged mice, which restored muscle regenerative capacity [8]. Chronic unweighted wheel running in aged mice also reshaped aspects of gene expression to reflect that of younger mice; this included genes related to lipid metabolic processes and TNF-α signaling specifically in myofibers determined by single fiber RNA-seq [8]. Collectively, these initial studies highlight that the complex molecular circuitry following exercise training is cell type-specific and influenced by age.

**Fig. 1:**
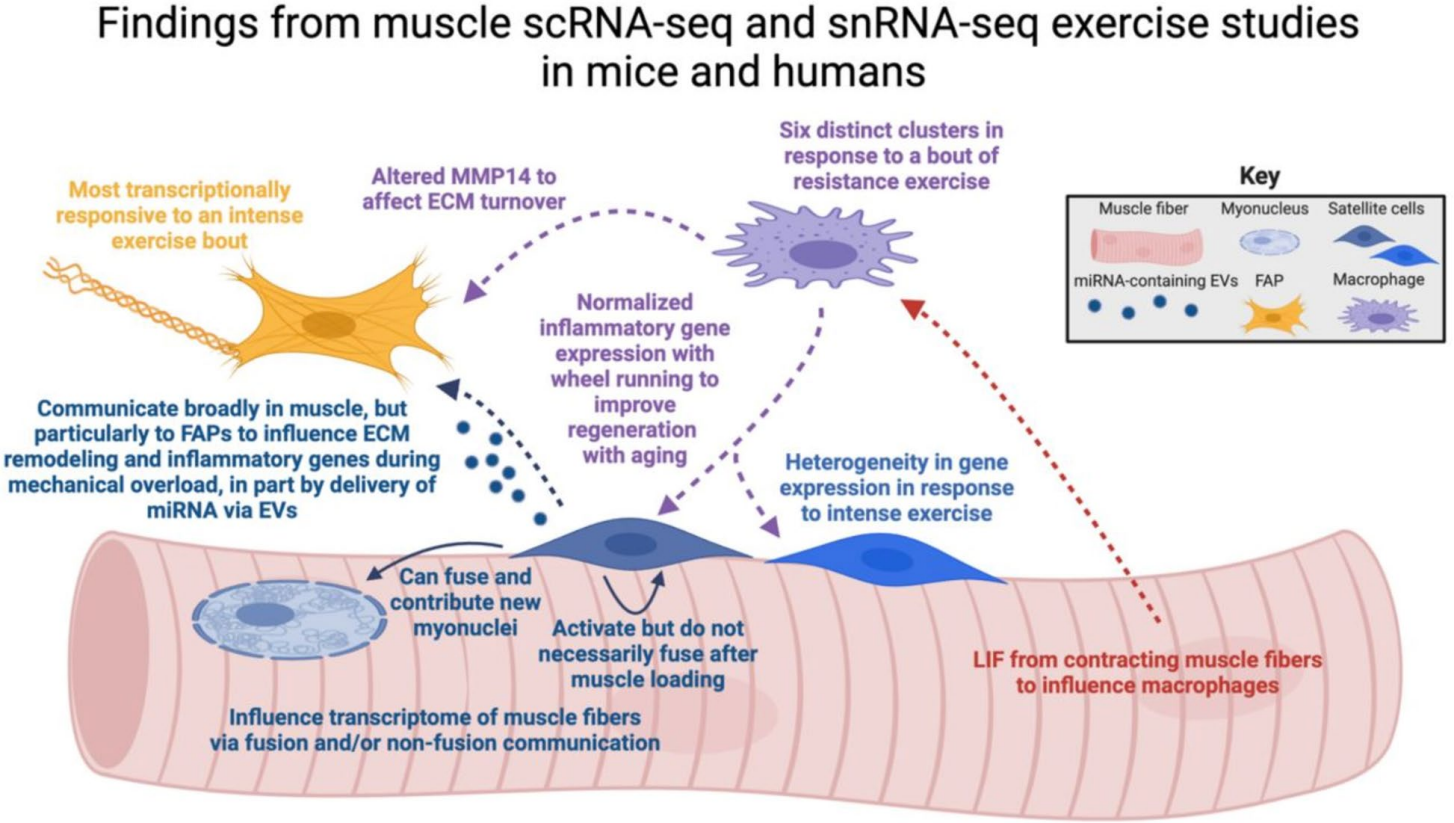
Summary and interpretation of findings outlined in this article from published scRNA-seq and snRNA-seq from skeletal muscle exercise studies in mice and humans. EVs, extracellular vesicles; FAP, fibro-adipogenic progenitor cell; LIF, leukemia inhibitory factor; MMP14, matrix metalloproteinase 14; miRNA, microRNA. Created with BioRender.com

In humans, scRNA-seq was recently used to explore muscle transcriptomic responses three hours following bouts of repeated high-intensity sprint exercise in two adult males and a female [9]. These analyses revealed a shift in mononuclear cell frequency—increased lymphocytes and monocytes and decreased proportion of endothelial cells—after intense exercise. Mesenchymal cells (likely FAPs) were most responsive to intense exercise, but most identified cell types that were profiled had an altered mRNA landscape. Activated satellite cells separated into distinct populations after exercise which may represent functional heterogeneity based on troponin gene expression (*TNNI1*: slow-twitch associated, or *TNNI2*: fast-twitch associated). Using scRNA-seq in an older male who previously mounted an effective hypertrophic response to a 14-week resistance exercise training program, Long et al. showed the heterogeneity of immune cells present in muscle 24 h following a single bout of high intensity resistance exercise [10]. These data broadly agree with the aforementioned murine exercise studies regarding mononuclear cells in muscle: macrophages, FAPs, and satellite cells are focal points in the muscle adaptive process. See Fig. 1 for an overview of the findings that we elaborated on in this manuscript.

## Future directions for single cell/nuclear -omics research with exercise in skeletal muscle

-Omics technology is evolving rapidly, expanding the capabilities of analysis at single cell and single nucleus resolution. These technologies are enabling new insights into cellular fate and interactions with exercise in skeletal muscle. Moving forward, it will be important to conduct acute and chronic exercise studies in rodents and diverse human populations across the lifespan with increasing sample sizes, as the cost of scRNA-seq continues to decline. It will also be vital to exploit snRNA-seq to gain a deeper understanding of the myofiber transcriptome with exercise, which is not the focus of scRNA-seq in muscle due to the syncytial nature and large size of muscle fibers. snRNA-seq will provide detailed information on how myonuclei interact to coordinate homeostasis and adaptation within the myofiber syncytium. The hope is that advances in sequencing technology will allow for deeper sequencing coverage per cell and more cells to be sequenced at an affordable price to provide an increasingly comprehensive picture of the transcriptome in different cell/nucleus populations. Furthermore, advances in artificial intelligence and deep learning have enabled inference-based computational techniques that predict cellular fate and intercellular interactions with surprising accuracy; these impactful technologies are likely to continue improving. In addition to the analysis of mRNA, interrogating non-protein coding RNA, small RNA, epigenetics (e.g., ATAC-seq, DNA methylation, histone modifications), proteomics, phosphoproteomics, and metabolomics at single cell and single nucleus resolution in muscle with exercise will further our knowledge of how exercise mediates its myriad of beneficial effects. These cell type-specific granular analyses will also help determine the underlying basis of exercise response heterogeneity so that personalized exercise programs can be designed and implemented. The skeletal muscle field is at the dawn of the single cell/nucleus -omics era. The next 20 years are sure to produce exciting new discoveries in muscle plasticity made possible by an understanding of cellular heterogeneity.

## Data Availability

Not applicable.
